# Joint care of parents and infants in perinatal psychiatry

**DOI:** 10.1192/j.eurpsy.2021.203

**Published:** 2021-08-13

**Authors:** A.-L. Sutter-Dallay

**Affiliations:** Univ. Bordeaux, Inserm, Bph, U1219, F-33000 Bordeaux, France And Perinatal Psychiatry Network-university Department Of Child And Adolescent Psychiatry, Bordeaux University and Charles Perrens Hospital, Bordeaux Cedex, France

## Abstract

**Abstract Body:**

In the perinatal period, about 15-20 % of women will present a mental health disorder. These disorders, as with all sources of psychological and physical stress in early childhood, especially the poor quality of parent-child relationships, are widely involved in predicting poor mental health in adulthood. The economic cost of perinatal mental health, corollary of this human cost, evaluated in 2014 would amount to £GBP 8.1 billion per annual birth cohort according to a UK report. This report highlights another fundamental element: 3/4 of the costs are associated with the deleterious consequences of parental psychological disorders on child development. The mechanisms involved in the relationship between parental psychiatric disorders and child development are complex. On the other hand, the influence of parental characteristics on the future of children can vary depending on social determinants such as familial income level. During the perinatal period, parental mental health represents one of the keys to the infant development. Perinatal psychiatry allows a dual approach essential to deal with the complexity of perinatal psychiatry care, combining a curative aim (care of the parent) and a preventive one (preventing the risk of dysfunction in the process of becoming parents, in parent-child relationships and of impaired child developement). This intervention wil discuss how this interactive circle must be supported by perinatal mental health policies, of which the joint care of parents and infants (from parent-child psychotherapy to joint mother-baby hospitalisation) in perinatal psychiatry is a pivotal element.

**Disclosure:**

No significant relationships.

